# Factor VIII: Perspectives on Immunogenicity and Tolerogenic Strategies

**DOI:** 10.3389/fimmu.2019.03078

**Published:** 2020-01-17

**Authors:** David W. Scott, Kathleen P. Pratt

**Affiliations:** Department of Medicine, Uniformed Services University of the Health Sciences, Bethesda, MD, United States

**Keywords:** factor VIII, tolerance, hemophilia, inhibitors, regulatory T (Treg) cells

## Abstract

Therapeutic treatment of bleeds with FVIII can lead to an antibody response that effectively inhibits its function. Herein, we review the factors that contribute to this immunogenicity and possible ways to overcome it.

## Introduction

Self-non-self-discrimination is one of the basic tenets of the immune system. The general failure to respond immunologically to antigens in our bodies is learned during ontogeny, as aptly recognized by Ray Owen in the 1940's in seminal studies with dizygotic cattle twins which shared hematopoietic cells during fetal development (1). Thus, these siblings failed to react to red blood cell antigens or skin grafts of their unrelated twin because their immune systems had learned that they must be “self” during ontogeny. This phenomenon of “actively acquired (immunologic) tolerance” was experimentally verified by the Nobel Prize-winning experiments of Billingham, Brent and Medawar in mice (2). This process is specific because responses to unrelated antigens remains intact.

What happens in the case of a patient who fails to express a given human protein during ontogeny and is then subsequently exposed? The classic case is hemophilia A and B, where patients lack all or part of the factor VIII (FVIII) or factor IX procoagulant proteins, respectively, and therefore have never “acquired” tolerance to that protein as “self.” Prophylactic or on-demand treatment of bleeds with recombinant or plasma-derived FVIII can lead to an antibody response to this human (but “foreign”) protein that effectively neutralizes or inhibits its function in the coagulation pathway; these antibodies are called “inhibitors.”

We discuss here some recent approaches, focusing on several developed in our laboratories, to characterize anti-FVIII immune responses and to promote durable peripheral tolerance to exogenously administered FVIII.

## Factor VIII Immunogenicity

Thus, while lack of tolerance (non-self) to FVIII explains its immunogenicity, there are other factors that potentially play a role. These are listed in Table 1 and are discussed below. Clearly, non-self-proteins tend to be recognized as foreign, as the process of c selection has not occurred. The developing immune system simply hasn't seen the T- and/or B-cell epitopes in the protein. Interestingly, FVIII is usually administered to patients intravenously (i.v.), a normally tolerogenic route to safely administer foreign antigens, yet it is highly immunogenic compared to many other therapeutic proteins, with approximately one in four patients developing a clinically significant inhibitor. Indeed, many foreign proteins that pass through the lymphatic or venous system into lymphoid organs are ignored, unless they provide additional signals or so-called adjuvanticity, often referred to as “danger” signals (3). This is because they lack properties that can stimulate the innate immune system, e.g., by interacting with Toll-like receptors (TLR) or through other innate immune processes either directly or indirectly. Efforts to demonstrate this property in FVIII have included culturing of human monocyte-derived dendritic cells (MoDCs) with FVIII, or thrombin-cleaved FVIII, or FVIII complexed with its carrier protein von Willebrand factor (VWF); interestingly, neither the maturation nor the T-cell stimulatory capacity of the MoDCs were affected (4). Skupsky et al. suggested that the biologic activity of FVIII in the clotting cascade, which leads to accelerated thrombin activation, provided an alternative mechanism of stimulating innate immune signaling (5). They found that treatment of mice with the anticoagulants warfarin or hirudin, which inactivate thrombin, reduced the immunogenicity of human FVIII in hemophilia A mice. In contrast, Meeks and co-workers, who engineered human FVIII proteins having several amino acid substitutions that neutralized its procoagulant activity, found that the immunogenicity of these non-active FVIII proteins was highly similar to that of active FVIII, thus leaving the role of its biological activity leading to immunologic “danger” as moot (6). If thrombin activation contributes to immunogenicity, then one might expect factor IX to also be unusually immunogenic when administered to hemophilia B patients. Inhibitor development in hemophilia B is actually rare, but this is likely due to the fact that most hemophilia B patients actually circulate a dysfunctional factor IX protein. They therefore could only respond to far fewer epitopes than patients with null mutations.

**Table 1:** Factors affecting the immunogenicity of human protein therapeutics.

**Product-related**	**Patient-related**
Self or non-self	HLA/genetics of patient
Presence of new (neo) epitopes	Route of administration
Biologic (enzymatic) properties	Underlying infection or pathology
Innate signaling properties	Immunosuppression
Absence of regulatory epitopes	Other medications
Formulation or aggregation	
Glycosylation (extent and type)	SNPs and other immunogenomic variants
Post-translational modifications including oxidation	
PEGylation or other protein engineering	
Organ and cell type (e.g., if delivered via gene therapy)	

Uptake and processing by antigen-presenting cells (APC) is the first step in the immune response to protein antigens. Proteolytic processing leads to presentation of peptides in major histocompatibility complexes on the APC surface, e.g., dendritic cells (DC). As noted above, peptides for which no thymic deletion has occurred may be immunogenic provided that they can be processed and presented on MHC Class I or Class II on mature DC, and that a T-cell receptor (TCR) on a circulating T cell recognizes and engages the resulting MHC-peptide complex. Interestingly, exposure to FVIII does not provoke a CD8^+^ immune response in hemophilia A patients or in murine FVIII^−/−^ mice, whereas CD4^+^ T-cell help (7) is essential for the development of high-titer anti-FVIII antibodies (8). MHC Class II peptide presentation provides “signal one” to effector CD4 T cells in the peripheral repertoire. In contrast, it has been proposed that many proteins may contain promiscuous peptide sequences that preferentially activate T *regulatory* rather than CD4 effectors; these have been termed “Tregitopes” (9, 10). These peptide sequences are commonly found not only in immunoglobulins but in many infectious agents, which may enable them to modulate and reduce the immune response to those agents. The potential role of Tregitopes in modulating FVIII immunogenicity, however, has not yet been established.

Last, but not least, is the physical properties of the FVIII antigen that may influence immunogenicity, such as post-translational modifications or physical aggregation, especially when the antigen is stored or administered at high concentrations. This may be due to an intrinsic or extrinsic property of the antigen, e.g., caused by charge changes, or by physical perturbations resulting from heating or formulation (11, 12). Differences in glycosylation patterns, e.g., according to the type of cell expression system, and covalent modifications to extend protein half-life (PEGylation, fusions of FVIII with other proteins or domains, etc.), and B-domain removal all could affect the immunogenicity of FVIII. The recent, prospective SIPPET study showed a significantly higher inhibitor incidence in previously untreated patients receiving a recombinant FVIII product, compared to plasma-derived FVIII (13). The biological basis for this difference remains to be identified.

Beyond the above properties, one must consider additional factors that influence immunogenicity which may be manifested in the recipients of FVIII replacement therapy. While there is no clear linkage to the HLA of the patient, HLA does affect which peptides will bind to the MHC on DC. Indeed, HLA Class II-restricted epitopes in FVIII were identified years ago by peptide proliferation assays (14–19). Subsequent isolation of FVIII-specific T-cell clones by classical limiting dilution (20) or by using HLA Class II tetramers loaded with FVIII peptides (7, 21–24) provided unambiguous identification of specific high-avidity epitopes (25). At the level of the repertoire, one must consider the nature of the mutation in the FVIII gene (*F8*) that leads to delayed or absent pro-coagulant activity: patients with a major deletion or other mutation precluding expression of the FVIII protein should lack tolerance to all of the epitopes in FVIII. On the other hand, those with missense mutations, which generally are associated with mild or moderate severity hemophilia A due to a partially disabled but still full-length FVIII protein, have a lower risk of developing an inhibitor response following FVIII infusions. In addition to FVIII mutations, other genetic factors, as well as environmental differences including “danger” resulting from trauma or surgery, influence the risk of hemophilia A patients developing an inhibitor (26, 27). Meunier et al. recently determined the frequency of FVIII-specific CD4^+^ T cells in the periphery of non-hemophilic blood donors and found approximately equal numbers of memory and naïve cells (28). Earlier studies had documented both FVIII-reactive antibodies (29, 30) and FVIII-specific T cells (16) in healthy control subjects. These studies demonstrated that FVIII is an unusually immunogenic self-protein, as also indicated by the rare autoimmune antibody response to FVIII known as “acquired hemophilia A.”

Several studies have suggested that hemophilia A patients with Black African or Hispanic ancestry experience a higher incidence of inhibitors, compared to white patients (31–33). There are multiple naturally occurring, non-hemophilia causing variants of the *F8* gene in the human population, including non-synonymous single nucleotide polymorphisms (ns-SNPs) that encode amino acid variants (34). Thus, it is conceivable that hemophilia A patients who express a dysfunctional FVIII protein, and are exposed to a therapeutic FVIII having a different amino acid sequence, could mount an immune response to the neo-epitope corresponding to this amino acid sequence (35). Although this is a plausible scenario, statistical analyses of inhibitor incidences in patients whose *F8* sequence at these sites was known (33, 36–38), as well as tetramer-guided epitope mapping to detect CD4^+^ T cells specific for these “mismatched” sequence (36), indicated that immune responses to these potential neo-epitopes occur rarely, if at all, and are therefore unlikely to contribute significantly to the immunogenicity of therapeutic FVIII.

FVIII is usually administered intravenously (i.v.), whereupon it rapidly binds to von Willebrand factor, which may modify its immunogenicity (39–41). The i.v. route is usually tolerogenic when infusing aggregate-free proteins into mice (42). This has been interpreted to suggest that i.v.-administered proteins fail to activate DC and to be processed in an immunogenic manner. However, in contrast to soluble proteins like ovalbumin, which is not immunogenic without adjuvant, FVIII is highly immunogenic when administered i.v. to the majority of FVIII knockout (E16) mice (5, 43, 44). Indeed, administering FVIII mixed with OVA can lead to an anti-OVA response, consistent with the intrinsic adjuvanticity of FVIII (5).

Finally, one has to consider other extrinsic properties of the host aside from HLA or other genetic factors. That is, an underlying infection will create significant inflammation which can tilt the response from tolerance to immunity. This would be a potential concern if a hemophilia A patient has an indwelling cannula which gets infected. On the other hand, a number of medications, especially steroids, are immunosuppressive and can tilt the immune response non-specific toward tolerance (45). Interestingly, both murine model studies and statistical analyses of patient outcomes indicate that immunizations do not affect inhibitor risk (46, 47).

The immunogenicity of FVIII that results in formation of inhibitors is a major impediment for the prevention and treatment of bleeds. While bypassing agents, including the FVIII-mimetic antibody emicizumab (48), or recombinant factor VIIa (49, 50), or FEIBA (Factor Eight Inhibitor Bypassing Agent, which is essentially a plasma-derived pro-coagulant protein cocktail) can facilitate clotting, are critically important lifesaving agents (51), they do not overcome the need to induce tolerance to FVIII. In particular, FVIII remains an essential component of the clinical armamentarium to support surgery, and to restore hemostasis following trauma, whereas the bypassing agents may be less efficient and/or carry a risk of thrombosis if doses are not carefully monitored. The relative risk/benefit ratios of utilizing FVIII vs. recently introduced novel bypass agents to control bleeding in specific clinical scenarios will become more apparent with further research and clinical “real world” experience.

## Modulation of FVIII Immunogenicity

Numerous methods to induce specific tolerance have been described for decades (52, 53). In terms of tolerance therapies to eradicate and prevent reoccurrence of inhibitors in hemophilia A patients, the standard clinical practice is intravenous repeated FVIII administration, which is called Immune Tolerance Induction (ITI). This protocol, first described by Brackmann and Gormsen in 1977 (54), is based on the high dose tolerance described by Mitchison in the 1960's (55) and essentially entails antigen overload, as well as maintaining higher trough levels of FVIII for continuous antigen exposure. This procedure is more often successful with patients having low tittered inhibitors but often fails in patients with higher titers. Moreover, it is expensive and challenging for patients and families, due to the need for frequent (often daily) infusions. Alternative methods to induce tolerance have primarily been tested in animal models, and most have not reached standard clinical practice. Below is a summary of several approaches in our labs, but it is not meant to be inclusive.

Following on the work of Weigle and colleagues (42, 56) with ultracentrifuged IgG as a model tolerogen, Borel utilized fusions or haptens and antigens on IgG carriers as tolerogens (57, 58), the latter being dependent on the presence of the IgG Fc fragment (9, 10). This would presumably crosslink the B-cell receptor with inhibitory Fc gamma receptors, an approach we will return to below. Based on the tolerogenicity of IgG fusions, we used retroviral transduction of FVIII domains with an IgG heavy chain in B cells as a tolerogenic protocol. This platform was successful in several autoimmune model systems as well (59–62); ironically, this approach was dependent on MHC class 2 presentation of peptides by B cells that led to the generation of regulatory T cells (Tregs) for both its induction and maintenance (63, 64).

Indeed, recent development of Fc fusions of clotting factors FVIII and FIX, designed for a longer half-life *in vivo* (65), have turned out to be tolerogenic in murine models and to induce Tregs (66, 67). This was initially supported by anecdotal cases reports of hemophilia A patients that suggest that FVIII-Fc is potentially tolerogenic (68–70); more highly powered clinical trials are in progress (NCT02234323, NCT03093480, and NCT03103542). Whether the tolerogenicity of Fc fusions is due to the regulatory epitopes in the constant region (9, 71) that turn on Tregs, and/or inhibitory Fc receptors (72, 73) is not clear.

While polyclonal human regulatory T cells (Tregs) have been proposed to treat autoimmune diseases and transplant rejection, and are already in clinical trials, the frequency of specific Tregs is very low. Moreover, the risk of non-specific immunosuppression and viral reactivation is real (74). Expansion of specific Tregs using peptide/APC and IL-2 has recently been achieved (75). Our labs have approached this issue by expressing specific receptors (or antigen) in expanded polyclonal Tregs or CD8 T cells, based on the seminal work by Eshhar (76, 77) and on clinical success of chimeric antigen receptor (CAR) T cells as reported by June and colleagues (78, 79). Since these studies have recently been published (80–83) and reviewed (52, 84), we will provide only a brief outline of these approaches to induce immune tolerance.

Starting with Tregs purified from healthy donors, our efforts to engineer specificity into polyclonal Tregs used retroviral transduction of specific T-cell receptors (TCR) (80) or CARs (scFv) (81), or even antigen (as B-cell Antibody Receptor = BAR) (83). In the first application, we cloned TCRs from FVIII reactive T-cell clones obtained from mild hemophilia patients (24). These clones recognized a peptide in the FVIII C2 domain restricted to HLA DRB1^*^01:01(21,22,24). The expanded TCR-transduced human Tregs suppressed proliferation and cytokine production by effector CD4 T cells even when the responders were in excess. Interestingly, the TCR-transduced Tregs also suppressed anti-FVIII B-cell responses *in vitro* and *in vivo* across a xenogeneic barrier (80)! Interestingly, although the engineered TCR recognizes a single peptide in the large FVIII protein, the antibody response to other major epitopes of FVIII was also suppressed. Thus, engineered FVIII-specific Tregs exhibit bystander suppression, an effect also seen with a TCR specific for a myelin peptide in a model of multiple sclerosis, an effect which appears to be due to uptake of IL-2 produced by effector T cells (85).

TCR-transduced Tregs are MHC class II restricted, thus limiting their eventual utility only to patients sharing the same HLA allele. Therefore, in the second approach, we collaborated with Anja Schmidt and Christoph Königs in Frankfurt, who provided a single chain Fv (scFv) that recognized the FVIII A2 domain. Like the CARs used in cancer therapy, these recognize conformational determinants and are not MHC restricted. Transduction of one of these scFv, called ANS8, into human Tregs also led to significant suppression of anti-FVIII responses *in vitro* and *in vivo*. Extensive dose response comparisons have not been performed as yet with these two types of engineered Tregs; the advantages (and disadvantages) of these specific Tregs are discussed elsewhere (Scott DW, *Molecular Therapy* submitted 2019).

Lastly, we hypothesized that it might be possible to directly target FVIII-specific B cells by expressing FVIII domains on the surface of Tregs. We refer to these as BAR Tregs, reflecting the fact that surface IgM/IgD on B cells react with these antigens. This was successfully achieved with both human BAR Tregs that expressed FVIII A2 and/or C2 domains and suppressed anti-FVIII responses *in vitro* and *in vivo* (83). The target of these BAR Tregs was proven to be the B cell, based on cell mixing experiments (83). Interestingly, in an allergy model, the target may also include sensitized mast cells, based on results of passive anaphylaxis experiments (86).

An alternative approach utilized transduced cytotoxic CD8 T cells expressing the targeted FVIII domains (82), as was done by Ellebrecht et al. with desmoglein 3 for possible therapy of pemphigus vulgaris, a devastating skin disease (87). [They refer to their antigen-expressing CD8 T cells as chimeric autoantigen receptor T cells (CAAR)]. Whether they are called BAR or CAAR, the cytotoxic T cells are highly specific and do not display bystander effects. There are situations when such specificity and lack of bystander effect might be necessary to carefully target part of a large antigen, but Tregs might be preferred if one doesn't know the targeted domains, and in the case of large multi-domain protein antigens such as FVIII.

Finally, a nanoparticle approach has been developed that can provide an alternative to engineered cellular therapies for tolerance; nanoparticles have also been used for drug delivery and vaccine development (88). Such nanoparticles can contain drugs such as rapamycin and are delivered with the target antigen (either attached or concomitantly) and presumably are taken up by dendritic cells, which act as tolerogenic APC and induce Tregs (89, 90). The use of rapamycin-containing nanoparticles for tolerance was successfully used by our group for FVIII (91), and by others for modulating autoimmunity (89) or the immune response to therapeutic immunotoxins, which are highly immunogenic (92, 93).

Several other approaches, in addition to the above strategies, are being developed to promote tolerance to FVIII. These include hepatic gene therapy, oral tolerance, and trans-placental delivery of FVIII. These are discussed more comprehensively in a recent review (84).

## Discussion

In conclusion, while there are multiple factors that influence the immunogenicity of therapeutic proteins, novels approaches such as those described here have the potential to modulate such immunogenicity. Time will tell which of these approaches may become cost-effective clinical therapies in the future.

## Author Contributions

DS and KP wrote and edited this manuscript.

### Conflict of Interest

The authors declare that the research was conducted in the absence of any commercial or financial relationships that could be construed as a potential conflict of interest.
